# Health-related quality of life and post-traumatic stress disorder in inpatients injured in the Ludian earthquake: a longitudinal study

**DOI:** 10.1186/s12955-020-01470-5

**Published:** 2020-07-14

**Authors:** Wanqiu Yang, Ke Cui, Timothy Sim, Jun Zhang, Yanchun Yang, Xiaohong Ma

**Affiliations:** 1 The Mental Health Center, Yunnan University, Kunming, 650091 People’s Republic of China; 2 School of Public Administration, Sichuan University, Chengdu, 610065 People’s Republic of China; 3 Department of Applied Social Sciences, The Hong Kong Polytechnic University, Hung Hom, Hong Kong; 4 The Mental Health Center, West China Hospital of Sichuan University, Chengdu, 610041 People’s Republic of China; 5 Psychiatric Laboratory and Mental Health Center, West China Hospital of Sichuan University, Chengdu, 610041 People’s Republic of China; 6 West China Brain Research Center, West China Hospital of Sichuan University, Chengdu, 610041 People’s Republic of China

**Keywords:** Injured inpatients, Posttraumatic stress disorder, Health-related quality of life, Disasters

## Abstract

**Background:**

The aim of this longitudinal study was to identify risk factors for posttraumatic stress disorder (PTSD) in inpatients injured in the Ludian earthquake and examine the relationship between PTSD symptoms and health-related quality of life (HRQoL) following the earthquake.

**Methods:**

Three assessments were performed during an 18-month follow-up period. In total, one-hundred forty-seven inpatients of one-hundred seventy-four inpatients (85% of the initial sample) underwent all the assessments. Injured inpatients admitted to the No. 1 People’s Hospital of Zhaotong City after a severe earthquake (6.5 on the Richter scale) were enrolled in the study and assessed using the Posttraumatic Stress Disorder Checklist-Civilian Version, Clinician-Administered Posttraumatic Stress Disorder Scale, and Medical Outcomes Study Short Form-36 Scale.

**Results:**

At the first, third and eighteenth months after the earthquake, the prevalence rates for PTSD were 23, 14, and 7%, respectively. In a regression model, bereavement, history of major diseases, and severe injury in the earthquake were associated with severe PTSD symptoms. HRQoL was negatively correlated with PTSD symptoms. Compared to that of Chinese norms, participants’ HRQoL was significantly lower in all eight HRQoL domains of the Medical Outcomes Study Short Form-36 Scale.

**Conclusions:**

The findings suggest that a substantial proportion of inpatients injured in the earthquake experienced severe PTSD symptoms and poor HRQoL. Therefore, early preventive programs and interventions should be implemented following disasters, to reduce PTSD and improve HRQoL in injured individuals.

## Background

Natural disasters, such as earthquakes, cyclones, floods, and tsunamis, lead to not only direct economic loss, death, and physical injury but also long-term adverse psychological outcomes [1]. A proportion of survivors experience long-term distress and psychopathology including posttraumatic stress disorder (PTSD), depressive disorder, and other disorders [2]. PTSD is the most prevalent of these adverse psychological outcomes [3, 4]. The consequences of disaster exposure, such as the presence of physical injury, fear of death, the loss of loved ones, and property loss, have been shown to be stronger predictors of PTSD relative to disaster type [4]. In addition, seriously injured trauma-exposed patients who require extended inpatient hospital admission could be at the greatest risk of PTSD development [5, 6].

The current literature suggests that approximately 10 to 40% of injured survivors will develop PTSD [5, 7, 8]. Some studies conducted after the Wenchuan earthquake in China reported that, 1 month after the disaster, the prevalence rates for PTSD in injured inpatients ranged from 17.1 to 45.9% [9–11]. Differences in PTSD prevalence rates between studies could result from differences in trauma types, degrees of trauma exposure, participant characteristics, the instruments used to measure PTSD, and the time from trauma to PTSD assessment [12, 13]. In China, PTSD studies involving survivors of natural disasters have focused mainly on hospitalization, particularly those examining the 2008 Wenchuan earthquake. However, few studies have explored long-term outcomes, such as 1–2 year or several years after disasters, for survivors who are at high risk of PTSD [14, 15]. Therefore, prospective follow-up of injured survivors is required to identify risk factors for PTSD.

PTSD is independently related to a broad profile of functional impairments and diminished quality of life (QoL) following injury [16, 17]. Reduced QoL has been observed after catastrophic events, such as natural disasters, and could be linked to material loss, somatic injury, and psychological distress [18, 19]. Health-related quality of life (HRQoL) is a narrow concept of QoL and pertains to the physiological, psychological, and functional aspects of well-being from the individuals’ own perspective [20]. Recent studies demonstrated that depression, anxiety disorders, and PTSD could exert a profound effect on HRQoL [21, 22]. Moreover, HRQoL is negatively correlated with PTSD in survivors of natural disasters [2, 22, 23]. The recognition of risk factors for PTSD is essential for the early identification of individuals at increased risk of experiencing these outcomes, to improve their HRQoL [24].

The present study aimed to provide insight into the prevalence, course, and predictors of PTSD and elucidate the relationship between PTSD and HRQoL in inpatients injured in the Ludian earthquake, via a longitudinal follow-up study, in contrast to most previous studies based on cross-sectional analysis.

## Methods

### Study setting

On August 3, 2014, an earthquake (6.5 on the Richter scale) occurred in Yunnan Province of China, with 617 deaths, 112 missing persons, 3143 injuries, and 1,088,400 victims. The epicenter was located in Zhaotong City, a medium-sized city located southwest of China. The region has experienced serious natural disasters in recent years and was hit by the YiLang earthquake (5.7 on the Richter scale) in 2012, the Ludian earthquake (6.5 on the Richter scale) in 2014, and a flood in 2016.

### Participants and procedures

**Fig. 1:**
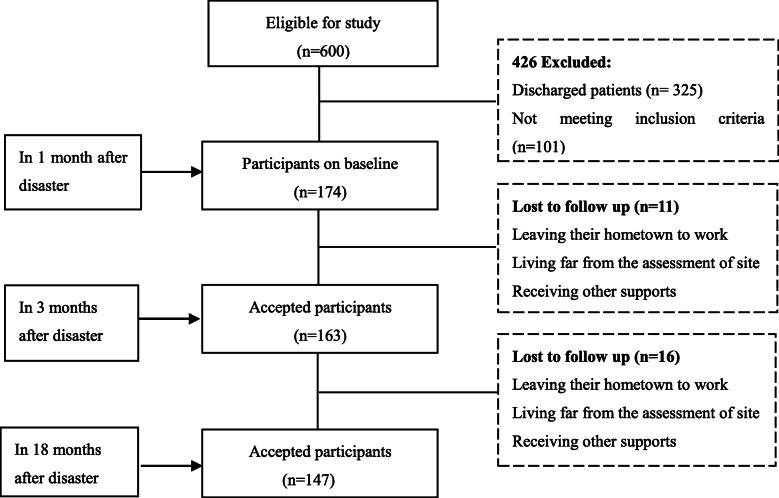
Flowchart of this study

This longitudinal study included injured inpatients in the Ludian earthquake and treated at the No. 1 People’s Hospital of Zhaotong City. Of approximately 600 injured patients, approximately 200 were discharged from the No. 1 People’s Hospital of Zhaotong City within a week, and 125 were transferred to other hospitals because of the severity of their injuries. In total, 174 patients aged between 10 and 75 years fulfilled the following inclusion criteria: (I) suffered physical injury caused by the Ludian earthquake, (II) treatmented in the hospital for longer than 1 month, (III) had no history of alcohol dependence or addictive drug use; and (IV) had no current pregnancy or breastfeeding; however, 101 injured patients did not meet the inclusion criteria for the study (shown in Fig. 1).

In total, 174 patients participated in the baseline assessment, and 163 and 147 participated in the first and second follow-up assessments, respectively. The initial assessment was conducted at the No. 1 People’s Hospital of Zhaotong between September and October 2014, approximately 1 month after the earthquake. The first follow-up assessment was conducted 3 months after the earthquake, between November and December 2014, and involved patient interviews at the hospital or resettlement sites, because most of patients had been discharged. Of the original 174 participants, 11 did not participate in the second assessment. The second follow-up assessment was conducted 18 months after the earthquake, in February 2016, in patients’ communities. Of the original 174 participants, 27 did not participate in the third assessment. A flowchart of the study is shown in Fig. 1.

The study was approved by the ethics committee at Sichuan University in Chengdou, China (2014) and was performed in accordance with the Declaration of Helsinki and its later amendments. All participants provided informed consent. Data were collected via face-to-face interviews conducted by four local psychiatrists and individuals who held master’s degrees in psychology from Sichuan university and were trained in group discussion and standardized data collection procedures. The four psychiatrists each held more than 5 years’ work experience and reviewed the Diagnostic and Statistical Manual of Mental Disorders, Fourth Edition (DSM-IV) diagnostic criteria for PTSD and the Clinician -Administered Posttraumatic Stress Disorder Scale (CAPS) prior to the interviews.

### Measures

Participants’ demographic characteristics, including age, sex, ethnicity, educational level, marital status, occupation, earthquake-related disaster exposure, and experiences after the Ludian earthquake were measured via a self-report questionnaire, which included the following questions: (1) Was your house destroyed entirely in the earthquake? (2) Were you buried or did you witness the death of a family member in the earthquake? (3) Did a family member die in the earthquake? (4) Were you with your family members when the earthquake occurred? (5) Have you been diagnosed with any major disease such as hypertension, stroke, heart disease, diabetes, or tumor? (6) Did you experience any major life events, such as the death of a family member (child, spouse, or parent), serious injury, disability, or divorce, before the earthquake? All these questions were coded dichotomously as yes/no items (shown in Self-report Interview Questionnaire 1).

### Post-traumatic stress disorder checklist-civilian version

The PTSD Checklist–Civilian Version (PCL-C) was used to measure self-reported trauma-related stress 1 and 3 months after the disaster. The PCL-C is a clinical self-assessment diagnostic scale consisting of 17 items that correspond to the fourth edition of criteria the Diagnostic and Statistical Manual of Mental Disorders (DSM-IV) [25]. Responses are provided using a five-point Likert-type scale ranging from 1 (not at all) to 5 (extremely), with higher scores indicating more severe posttraumatic stress. Total symptom severity scores range from 17 to 85, and a cut-off point of 50 was recommended for the diagnosis of PTSD in Chinese sample [26, 27]. With a cut-off point of 50, sensitivity was 0.78, specificity was 0.86, and diagnostic efficiency was 0.83. The PCL-C scores were strongly correlated with the Clinician -Administered PTSD Scale (CAPS) scores. The correlation coefficient was 0.93, and diagnostic efficiency was 0.90 [28, 29]. The PCL-C has been used widely in various Chinese populations and has shown high internal consistency, with Cronbach’s α of 0.96 [30, 31].

### Clinician -Administered Posttraumatic Stress disorder Scale (CAPS)

Participants underwent a structured clinical interview via the CAPS, which was based on 1994 DSM-IV Diagnoses (American Psychiatric Association) [32], 18 months after the earthquake. The CAPS is recognized as one of the gold standards for the diagnosis of PTSD [33]. (The CAPS includes 30 items, with responses provided using a four-point Likert-type scale ranging from 1 (never) to 4 (routinely). The CAPS includes Criterion A (exposure to a traumatic event), Criteria B–D (core symptom clusters of re-experiencing, numbing, avoidance, and hyperarousal), Criterion E (chronology), Criterion F (functional impairment), and associated guilt and dissociation and assesses current and lifetime PTSD symptom status. The scale includes three subscales: re-experiencing, avoidance, and hyperarousal. Total scores range from 0 to 136, and patients with CAPS scores exceeding 65 are categorized as having PTSD, while those with CAPS scores exceeding 80 are categorized as having extreme PTSD [32]. The CAPS has shown good reliability and validity in Chinese samples [34, 35]. Moreover, Li et al. reported Cronbach’s αs between 0.80 and 0.90 and convergent validity of0.70 for the Chinese version of the CAPS [33, 35].

All assessments performed at 1 month (*N* = 174) and 3 months (*N* = 163) after the disaster involved the PCL-C, while those performed at 18 months (*N* = 147) after the disaster involved the CAPS, administered by four psychiatrists to confirm PTSD diagnosis. To evaluate investigator reliability, we selected 20 of the 147 cases at random for independent assessment by the four psychiatrists. Two of the 20 cases showed inconsistency in the diagnoses formed by the four psychiatrists. Therefore, we concluded that investigator reliability was 90%, and the diagnoses formed by the four psychiatrists showed good inter-rater reliability (*W* = .93, χ^2^ = 32.45, *P* < .001). It reasonable to speculate that the clinical diagnosis of patients at 18-month are more valuable. Thus, the results of 18-month were reported in detail.

### The medical outcomes study 36-item short form health survey

The Medical Outcomes Study 36-Item Short Form Health Survey (SF-36) is widely used in HRQoL research and has been validated and tested for reliability in some studies and used extensively to evaluate functional and QoL outcomes in injured individuals [6, 18]. The scale consists of two dimensions: the physical component summary and the mental component summary. The physical component summary contains four subscales: physical functioning (PF); role physical (RP), which pertains to role limitations because of physical health problems; bodily pain (BP); and general health (GH). The mental component summary contains four subscales: vitality (VT); social functioning (SF); role emotional (RE), which pertains to role limitations because of emotional problems; and mental health (MH) [36]. Subscales are scored from 0 to 100, and higher scores indicate greater HRQoL. The Chinese version of the SF-36 is used widely in China, and Cronbach’s αs for the scale have been reported between .70 and .80, with convergent validity ranging from .66 to .94 [37, 38].

### Statistical analysis

All data were analyzed using SPSS for Windows (version 16.0). The results of the descriptive analyses are presented as central tendencies for continuous variables and frequencies for categorical variables. The data for scores of the PCL-C, the CAPS and the SF-36 all passed the normality test. We performed t tests for continuous data and 2-sided Fisher exact test for categorical data, to compare differences between two groups (with PTSD and without PTSD at 18 months). Single sample t-test was used to compare the difference between patients and Chinese norms on the SF- 36 scale. The prevalence rates for PTSD were described as a percentage and the 95% confidence intervals for PTSD prevalence rates were calculated using binomial the distribution of these rate. The multivariate logistic regression analysis was performed to identify the independent effects of the predictors of PTSD (dependent variables). In all tests, coefficient values, odds ratios (ORs), and 95% confidence intervals (CIs) were used to quantify correlation strength. All tests were 2-tailed, and statistical significance was set at *P* ≤ .05.

## Results

### Demographic data and disaster exposure characteristics

Table 1 shows the participant data collected at 1 month, 3 months, and 18 months. Of the 174 patients assessed 1 month after the earthquake, 74 were men, 100 were women, 103 were married, 55 were unmarried, 16 were divorced, and 140 were Han, who is main ethnic group of the local area. The patients’ mean age (± sd) was 46 ± 18.4 years old, and their ages ranged from 10 to 88 years. The numbers of patients who had completed primary school, middle school, and high school and above were 56, 39, and 19, respectively, and 60 patients were illiterate. Most of the participants were peasants (*n* = 154). With respect to disaster exposure, 49 of 174 patients had experienced burial and bereavement, 24 patients were separated from their families, and 23 patients were affected by severe injury. The numbers of patients who reported a history of major diseases and life events were 27and 52, respectively.

**Table 1:** Demographic data and disaster exposure characteristics of the injured in 3, 6 and 18 months

	Variable	1 month n(%) (***N*** = 174)	3 months n(%) (***N*** = 163)	18 months n(%) (***N*** = 147)
Age		46 ± 18.4	45 ± 18.3	46 ± 17.6
Gender	Male	74 (43)	74 (45)	67 (46)
	Female	100 (57)	89 (55)	80 (54)
Ethnic group	Han	165 (95)	156 (96)	140 (95)
	Non-Han	9 (5)	7 (4)	7 (5)
Education	Illiteracy	60 (35)	55 (34)	46 (33)
	Primary school	56 (32)	54 (33)	54 (37)
	Middle school	39 (22)	37 (23)	37 (25)
	High school and above	19 (11)	17 (10)	10 (5)
occupation	Peasant	154 (89)	143 (88)	132 (90)
	Others	20 (11)	20 (12)	15 (10)
Marital status	Single	55 (32)	55 (34)	47 (32)
	Married	103 (59)	93 (57)	86 (59)
	Divorced	16 (9)	15 (9)	14 (9)
House collapsed completely	NO	58 (33)	47 (29)	39 (27)
	YES	116 (67)	116 (71)	108 (73)
Buried experience	NO	125 (72)	119 (73)	110 (75)
	YES	49 (28)	44 (27)	37 (25)
Bereavement	NO	125 (72)	119 (73)	110 (75)
	YES	49 (28)	44 (27)	37 (25)
History of major diseases	NO	147 (85)	140 (86)	124 (84)
	YES	27 (15)	23 (14)	23 (16)
Separation from family	NO	150 (86)	144 (88)	134 (91)
	YES	24 (14)	19 (12)	13 (9)
Major life events before the disaster	NO	122 (70)	116 (71)	104 (71)
	YES	52 (30)	47 (29)	43 (29)
Severe injury	NO	23 (13)	22 (14)	18 (12)
	YES	152 (87)	141 (86)	129 (88)

### Analysis of dropout

As shown in Table 1 and Fig. 1, during follow-up, 11 (6%) and 27 (16%) of participants did not attend the 3-month and 18-month evaluations, respectively. We analyzed the participants’ circumstances before dropout, to clarify the effect of missing data and explore the causes of dropout. Specifically, we used t tests to compare PCL-C scores between the dropout and follow-up groups. Baseline PCL-C scores for participants who did not attend follow up at 3 months (*n* = 11) did not differ significantly from that of participants who attended follow-up assessment (*n* = 163; 41 ± 11.5 versus 40 ± 13.7, *P* > .05). In addition, of the participants who attended follow up at 3 months, PCL-C scores for those who did not attend follow up (*n* = 16) at 18 months did not differ significantly from that of participants who attended follow-up assessment (*n* = 147; 31 ± 14.9 versus 38 ± 13.7, *P* > .05). Of the participants who dropped out, 74% (20/27) were men and 26% (7/27) were women. Of the participants who dropped out, 7% (2/27) were seriously injured and 93% (25/27) were not seriously injured.

Moreover, to understand the reasons for dropout, we randomly selected eight of the 27 participants who had dropped out and interviewed them by telephone to ask about their recovery status and the reasons why they did not wish to participate in the assessments. Three main reasons were reported: some participants had left their settlements or homes to work in other cities; some participants lived far from the assessment of site, which was approximately 4 h’ walk away; and two participants had begun to receive financial and health support from other social organizations. Therefore, it is reasonable to speculate that there were valid reasons for attrition. Most men who dropped out had left to work in larger cities. Further, some participants lived in scattered areas and most lived high in the mountains, and it was difficult for them to attend assessments at community health centers. In addition, both follow-up interviews were conducted in autumn and winter; therefore, it was difficult for participants to attend. Moreover, the rehabilitation assessments might not have provided sufficient support to patients, and participants received more support from the government or other social organizations.

### The prevalence of PTSD at 1 month, 3 months, and 18 months after the earthquake

The prevalence rates for PTSD assessed by PCL-C at 1 month and 3 months after the earthquake were (40/174) 23%(95% CI: 17%, 29%)and (22/163)14%(95% CI: 7%, 16%)respectively. Further, the prevalence of PTSD was (10/147) 7%(95% CI: 3%, 10%)at 18 months, according the criteria of PTSD (CAPS Scale). Therefore, the overall prevalence of PTSD decreased gradually following the earthquake. In addition, we checked the individuals with PTSD at 3-month and 18-month. Seventeen of twenty-two individuals who had PTSD at the 3-month assessment were screened positive-PTSD at the 1-month and eight of twenty-two individuals were still diagnosed with PTSD at the 18-month. Meanwhile, seven and eight of ten individuals who had PTSD at the18-month assessment were screened positive-PTSD at the 1-month and 3-month, respectively.

### Comparison of demographic characteristics and disaster exposure between the PTSD and non-PTSD groups at 18 months

The differences in demographic characteristics between the PTSD (*n* = 10) and non-PTSD (*n* = 137) groups 18 months after the disaster are summarized in Table 2. There were no significant differences between the two groups, according to age, gender, educational level, ethnicity, occupation, or marital status. In addition, the two groups did not differ significantly according to the consequences of disaster exposure, such as houses collapsing completely and separation from family, or the experience of major life events before the disaster. However, the proportions of participants who had experienced being buried 7/10 (70%) versus 30/137 (22%) (*P* = 0.003), being bereaved 7/10 (70%) versus 39/137 (29%) (*P* = 0.011), a history of major diseases 7/10 (70%) versus 16/137 (12%) (*P* < 0.001), and sustained severe injuries 7/10 (70%) versus32/137 (23%) (*P* = 0.004) in the PTSD group were significantly higher than those observed in the non-PTSD group. Moreover, the proportion of participants in the PTSD group who had undergone physical rehabilitation at 18 months was significantly lower than that observed in the non-PTSD group 6/10 (60%) versus 122/137 (89%) (*P* = 0.048).

**Table 2:** Demographics and disaster exposure of the injured with and without PTSD at 18 months post-disaster by univariate analyses

Demographic	Characteristics	Non-PTSD group n(%) (***N*** = 137)	PTSD group n(%) (***N*** = 10)	***P***-value
Age		46 ± 17.9	44 ± 14.8	0.094
Gender	male	63 (46)	4 (40)	0.755
	female	74 (54)	6 (60)	
Ethnic group	Han	130 (95)	10 (100)	> 0.999
	Non-Han	7 (5)	0	
Education level	Illiteracy	44 (32)	2 (20)	0.661
	Primary school	49 (36)	5 (50)	
	Middle school	35 (26)	2 (20)	
	High school and above	9 (6)	1 (10)	
occupation	Peasant	122 (89)	10 (100)	0.599
	others	15 (11)	0	
Marital status	Single	45 (33)	2 (20)	0.493
	Married	78 (57)	8(80)	
	Divorced	14 (10)	0	
Houses collapsed completely	No	37 (27)	2 (20)	> 0.999
	Yes	100 (73)	8 (80)	
Buried experience	No	107 (78)	3 (30)	0.003
	Yes	30 (22)	7 (70)	
Bereavement	No	98 (72)	3 (30)	0.011
	Yes	39 (28)	7 (70)	
History of major diseases	No	121 (88)	3 (30)	0.000
	Yes	16 (12)	7 (70)	
Separation from family	No	126 (92)	8 (80)	0.217
	Yes	11 (8)	2 (20)	
Major life events before the disaster	No	97 (71)	7 (70)	> 0.999
	Yes	40 (29)	3 (30)	
Severe injury	No	105 (77)	3 (30)	0.004
	Yes	32 (23)	7 (70)	
Rehabilitation at 18 months	No	15 (11)	4 (40)	0.026
	Yes	122 (89)	6 (60)	

### Risk factors for 18-month PTSD after earthquake

The results of the logistic regression to identify factors that predicted PTSD were as follows (Table 3). All significant variables (*P* < .05) in the univariate analyses were included in the logistic regression model to identify the independent role of each predictor variable after adjustment, including bereavement, a history of major diseases, severe injury, experience of being buried and rehabilitation at 18 months. The results of the logistic regression analysis indicated that risk factors for PTSD included bereavement (OR = 29.26, CI: 3.125–274.046), a history of major diseases (OR = 15.92, CI: 1.850–136.934), and severe injury (OR = 0.039, CI: 0.005–0.309). The experience of being buried (OR = 1.323, CI: 0.123–14.20) and rehabilitation at 18 months (OR = 0.144, CI: 0.019, 1.115) were not risk factors for PTSD.

**Table 3:** Relationship between main variables and PTSD analyzed by logistic regression

Variables		OR (95% CI)	***P***-value
Buried experience	NO	1	
	YES	1.323(0.123 ~ 14.200)	0.817
Bereavement	NO	1	
	YES	29.263(3.125 ~ 274.046)	0.003
History of major diseases	NO	1	
	YES	15.916(1.850 ~ 136.934)	0.012
Severe injury	YES	1	0.002
	NO	0.039(0.005 ~ 0.309)	
Rehabilitation at 18 months	YES	1	0.063
	NO	0.144(0.019 ~ 1.115)	

*Abbreviations*: *CI* confidence interval, *PTSD* posttraumatic stress disorder

### Assessment of HRQoL 18 months after the earthquake

There were differences in health status between the participants (*N* = 147) and the general Chinese population (*N* = 4251). In our study, 46 and 54% of patients was for male and female and the average age (± sd) was 46 ± 17.6 years, old, while among the 4251 respondents of the general Chinese population, male and female respondents accounted for 51% (2154) and 49% (2097) and the average age (± sd) was 41 ± 17.3 years old, ranging from 14 ~ 99 years old [36, 39]. The two groups differed significantly in all eight subscales of the SF-36 are summarized in Table 4. The participants’ mean subscale scores were significantly lower relative to those observed in the general Chinese population (BP: 72.10 ± 17.38 versus 83.3 ± 19.7, *P* < .001; PF: 63.80 ± 31.75 versus 87.6 ± 16.8, *P* < .001; RP: 11.14 ± 30.19 versus 83.0 ± 20.7, *P* < .001; GH: 47.5 ± 15.23 versus 68.2 ± 19.4, *P* < .001; VT: 48.26 ± 11.49 versus 70.1 ± 16.8, *P* < .001; SF: 78.26 ± 19.42 versus 84.8 ± 16.6, *P* = .001; RE: 23.19 ± 42.15 versus 85.3 ± 17.7, *P* < .001; MH: 57.00 ± 8.93 versus 78.8 ± 15.4, *P* < .001). These data indicated that the health status of inpatients injured in the earthquake was poorer relative to that of the general Chinese population.

**Table 4:** Comparison of HRQoL mean scores among the injured and general Chinese at 18 months post-disaster

	The injured (Mean ± SD) (***N*** = 147)	The Chinese norm (Mean ± SD) (***N*** = 4251)	***t***	***P***-value
Physical health domains				
Bodily Pain (BP)	72.10 ± 17.38	83.3 ± 19.7	−6.245	0.000
Physical Functioning (PF)	63.80 ± 31.75	87.6 ± 16.8	−6.917	0.000
Role-Physical (RP)	11.14 ± 30.19	83.0 ± 20.7	−22.822	0.000
General Health (GH)	47.5 ± 15.23	68.2 ± 19.4	−9.635	0.000
Mental health domains				
Vitality (VT)	48.26 ± 11.49	70.1 ± 16.8	−18.225	0.000
Social Functioning (SF)	78.26 ± 19.42	84.8 ± 16.6	−3.411	0.001
Role-Emotional (RE)	23.19 ± 42.15	85.3 ± 17.7	−14.135	0.000
Mental Health (MH)	57.00 ± 8.93	78.8 ± 15.4	−23.423	0.000

### Relationship between PTSD and QoL domains 18 months after the earthquake

The results of the analysis of correlations between PTSD and the domains of HRQoL showed that CAPS scores were negatively correlated with scores for all eight SF-36 subscales including BP, PF, RP, GH, VT, SF, RE, and MH. The correlation coefficients ranged from −.26 to −.53, as shown in Table 5.

**Table 5:** Relationship between PTSD and domains of RHQoL at 18 months post-disaster by correlation analysis

	***r***	***P***-value
Physical health domains		
Bodily Pain (BP)	−0.372	< 0.0001
Physical Functioning (PF)	−0.529	< 0.0001
Role-Physical (RP)	−0.320	< 0.0002
General Health (GH)	−0.448	< 0.0001
Mental health domains		
Vitality (VT)	−0.422	< 0.0001
Social Functioning (SF)	−0.482	< 0.0001
Role-Emotional (RE)	−0.255	0.014
Mental Health (MH)	−0.325	0.002

## Discussion

In this longitudinal study, we examined PTSD in inpatients injured in the Ludian earthquake. The prevalence rates for PTSD 1, 3, and 18 months after the earthquake were 23, 14, and 7%, respectively. Gao et al. reported that the prevalence rates for PTSD in survivors of bodily injury 1 month after the Wenchuan earthquake ranged from 35.56 to 45.90% [9, 10]. Moreover, Wang et al. found that, 3 months after the disaster, the prevalence rate for PTSD was 18.8% and the PTSD prevalence rate was 7.2% [40]. In addition, Zatizck et al. reported that more than 20% of injured trauma survivors displayed PTSD symptoms 12 months after acute care inpatient hospitalization in the USA [6], and Shalev et al. showed that the incidence rate for PTSD in inpatients 6 months after the disaster was 25.5% [5]. Several factors could have contributed to relatively low PTSD prevalence rate observed in the current study. For example, the development of PTSD is related to the characteristics of the trauma and the extent of physical injury [41], and more severe trauma generally exerts a greater impact on survivors relative to less severe trauma [5]. In addition, differences between PTSD screening instruments and their associated cut-off values increase the risk of assessment bias and reduce the reliability of prevalence estimates. Most studies have used a cut-off score of 41 for the PCL-C, while we used the recommended cut off score of 50 [24, 25]. Moreover, the current study used the CAPS to assess PTSD 18 months after the disaster, unlike previous studies.

Longitudinal studies are essential in understanding psychopathology following disasters. The reduction in the prevalence rate for PTSD from 23 to 7% in the current study was greater relative to that of the natural progression of untreated PTSD. One factor that could have contributed to the recovery observed in the current study was the provision of efficient medical and emergency assistance in response to the Ludian earthquake, based on lessons learned from the 2008 Wenchuan earthquake [42]. Another reason for this finding could be that, after the earthquake, psychological rescue teams were dispatched to the disaster area immediately to provide psychological services for injured hospital patients for approximately 3 months [43]. However, we cannot rule out the possibility that some participants recover from PTSD naturally without treatment, and it is important to determine whether early treatment leads to better outcomes relative to those observed with delayed or no treatment.

Multiple risk factors for PTSD in injured adults have been investigated [5, 6, 41, 44]. In the current study, we identified three risk factors for PTSD, including bereavement, history of major diseases and severe injury, but we did not find significant relationships between PTSD and demographic characteristics, such as previous life events, or property damage. An overwhelming majority of studies examining post-disaster PTSD have used cross-sectional study designs and reported substantially heterogeneous results [45]. However, the current study was longitudinal and included assessments at 1, 3, and 18 months after the disaster. The severity of injury was an important factor influencing PTSD symptoms [5, 7], and survivors who reported both injury and bereavement showed a significantly higher risk of PTSD relative to those who had not experienced injury or bereavement [7, 46]. Injury severity, bereavement, and history of major diseases could exert stronger effects on PTSD development relative to those exerted by demographic characteristics; however, future in-depth studies with larger samples are required to confirm these relationships.

To our knowledge, this was the first study to examine the relationship between PTSD and HRQoL in injured inpatients after a natural disaster in China. Many studies have shown that greater PTSD severity was associated with poorer psychosocial and physical HRQoL [2, 3, 39]. In the current study, HRQoL in injured survivors across both mental and physical dimensions was negatively correlated with PTSD. It is understandable that PTSD symptoms, such as intrusive thoughts, hypervigilance and nightmares, could affect social function and satisfaction with life. However, physical problems, such as pain and disability result in role limitations, and this could lead to emotional problems, which constitute known psychiatric impairment [40, 47]. Therefore, the physical dimensions of HRQoL could affect PTSD symptoms. Moreover, PTSD symptoms and HRQoL interact mutually, as other life events, such as divorce and job loss, worsen PTSD symptoms [47]. Therefore, future studies should focus on the long-term mutual interaction between PTSD and HRQoL following disasters.

In the current study, participants’ SF-36 scores in all eight QoL domains 18 months after the disaster were significantly lower relative to the Chinese norm-based score. Norm-based scoring of SF-36 scales is recommended to facilitate the interpretation of results across measures [48]. The greatest differences between the participants’ scores and the Chinese norm-based score were found in RP, GH, VT, RE, and MH, with the means of these subscales all below 50 in the current study. Low SF-36 scores indicate that people find it difficult to make use of social support [49]. However, social support is vital for mental health, and negative social support could increase the risk of PTSD development [50]. Therefore, the current findings suggest a need for psychological intervention and the provision of social support for inpatients after disasters.

## Conclusion

Exposure to trauma, such as bereavement, a history of major diseases, and severe injury could exert negative effects on patients injured in earthquakes and increase the long-term risk of PTSD. Moreover, HRQoL was negatively correlated with PTSD symptoms in inpatients injured in the earthquake in the current study. In addition, the participants’ levels of HRQoL were significantly lower relative to those of the general Chinese population. In future research, it would be appropriate to design and evaluate early interventions for implementation following disasters, to reduce PTSD symptoms and improve QoL for injured inpatients.

### Study limitations and strengths

The study was subject to some limitations. First, although PCL-C scores are very strongly correlated with CAPS scores, differences between the results obtained via these diagnostic tools for PTSD remain. In the comprehensive assessment of PTSD, the CAPS is one of the gold standards for use in PTSD diagnosis. In the current study, we used the PCL-C 1 and 3 months after the earthquake and the CAPS 18 months after the earthquake, because the PCL-C involves self-assessment and saves time, while the CAPS is used by psychiatrists and takes approximately 33 ± 16 min to complete [33]. In the early post-disaster period, when responders are faced with heavy rescue tasks and a shortage of psychiatrists, the PCL-C is used as an evaluation tool to screen inpatients quickly. In addition, among the ten patients with PTSD at 18 months in our current study, seven and eight patients were positive PTSD at 1 month and 3 months, respectively, and only one patient was not screened for PTSD at 1 month and 3 months, respectively. This may indicate a relatively high degree of consistency between the PCL-C and the CAPS for assessment PTSD. Therefore, the above-mentioned limitation should be noted in future research and confirmed in future investigations. Second, self-report measures, such as the PCL and SF-36 used in the study, could involve potential information bias. In fact, response bias occurs in many areas of behavioral and medical research in which self-reported data are used [51]. Actually, the “response shift bias” phenomenon is a source of contamination in self-report measures, which can lead to inaccurate test ratings [52]. Moreover, our study sample size is relatively small. Therefore, the current findings should be confirmed in large samples in future studies. Third, another limitation was the high attrition rate in the follow-up research. One of the reasons for this was that some participants had moved to larger cities to work. Another reason was that it was difficult for participants to attend the assessment site in the community health center. Further, caution should be exercised when generalizing the results to other countries, different cultures, and different disaster scenarios. Finally, comparisons with Chinese general population data were limited to global mean testing since detailed data by gender and age categories or other confounders were not available. Therefore, although crude absolute SF-36 scale differences varied from 6 (social functioning) to 72 (role physical) and were all statistically significant (*p* ≤ 0.001) the present results should be considered with caution. Despite these limitations, this was the first study conducted in China to examine QoL and explore PTSD via follow-up research involving inpatients following a disaster. In addition, the findings could contribute to the design of evidence-based interventions to promote QoL and reduce the prevalence of PTSD in injured inpatients.

## Supplementary information

**Additional file 1.** Investigation of Earthquake Impact.

## Data Availability

The datasets used and analyzed in the current study are available from the corresponding author on reasonable request.

## Abbreviations

QoL: Quality of life
HRQoL: Health-related quality of life
PTSD: Posttraumatic stress disorder
PCL-C: Post-Traumatic Stress Disorder Checklist-Civilian Version
CAPS: Clinician-Administered Posttraumatic Stress Disorder Scale
SF-36: Medical Outcomes Study 36-Item Short Form Health Survey
DSM-IV: Diagnostic and Statistical Manual of Mental Disorders, Fourth Edition
