# Supplementary material for: Health-related quality of life and post-traumatic stress disorder in inpatients injured in the Ludian earthquake: a longitudinal study
Source: Health Qual Life Outcomes. 2020 Jul 14;18:229. doi: 10.1186/s12955-020-01470-5 (PMC7362470; doi:10.1186/s12955-020-01470-5)
Supplement: Supplementary file 1 — Additional file 1. Investigation of Earthquake Impact. [file 12955_2020_1470_MOESM1_ESM.docx]

**Investigation of Earthquake Impact**

DIRECTIONS: This interview contains statements about some impacts of the “8.03 earthquake” on you, and some of these experiences may bring you discomfort. If you feel uncomfortable, you can slow down and discuss with our interviewers; if you do not want to continue to answer these questions, you can stop at any time. At the same time, if you have any questions or do not understand these questions, please let us know.

Code: name ： gender：

age： occupation:

Ethnic group: ① Han ②other (specific description)

Marital status: ①Single ②Married ③ Divorced

Education level: ①Illiteracy ②Primary school ③Middle school

④High school and above

1. Was your house destroyed entirely in the earthquake?

① Yes ②No

2. Were you buried or did you witness the death of a family member in the earthquake?

① Yes (specific description) ②No

3. Did a family member die in the earthquake?

① Yes (specific description) ②No

4. Were you with your family members when the earthquake occurred?

① Yes (specific description) ②No

5. Have you been diagnosed with any major disease such as hypertension, stroke, heart disease, diabetes, or tumor?

① Yes (specific description) No

6. Did you experience any major life events, such as the death of a family member (child, spouse, or parent), serious injury, disability, or divorce, before the earthquake?

① Yes (specific description) No

Thank you for your cooperation!

Health Research Group of Sichuan University

August 2014
